# Notes and Notices

**Published:** 1888-11

**Authors:** 


### NOTES AND NOTICES.

MARRIED.—Clark—Champion—October 23d, in Germantown, Pa., by the Rev. Dr. Faulkner, rector of Christ Church, George H Clark, M. D, to Alice Feinour Champion, daughter of the late John B. Champion. Our best wishes and congratulations.

A GOOD SURGICAL JOURNAL.—The *Annals of Surgery*, published by Messrs. J. H. Chambers & Co., of St. Louis, is the only English journal devoted entirely to surgical work. The great attention now given to surgery renders such a journal a necessity.

Drs. L. S. Pilcher (of Brooklyn) and C. B. Keetley (of London), are the Editors-in-chief and are assisted by many of the ablest surgeons of America and Europe, which is a sufficient guarantee of the journal's merit. Those who are interested in the study of surgery can hardly afford to omit *The Annals*.

LIBRARY OF DR. LIPPE.—We call attention of our readers to the following notice of the executor of Dr. Lippe's estate. It is a good opportunity to acquire some valuable homœopathic works.

ESTATE OF ADOLH LIPPE, M. D. (DECEASED).—The Medical Library and Medicines belonging to the above estate are offered for sale and proposals are solicited for the purchase of the whole or any part of either.

A catalogue of the Library will be sent to parties desiring to purchase upon application to

W. C. HALL, Executor.

251 South Fourth Street, Philadelphia.

